# 2025 Acknowledgment of JB *Ad Hoc* Reviewers

**DOI:** 10.1128/jb.00014-26

**Published:** 2026-05-21

**Authors:** George A. O’Toole

**Affiliations:** 1 Department of Microbiology and Immunology, Geisel School of Medicine at Dartmouth, Hanover, New Hampshire, USA

## EDITORIAL

Every year, *ad hoc* reviewers make critical contributions to the *Journal of Bacteriology* by reviewing papers in their field of expertise. This year, we acknowledge 518 such reviewers who reviewed at least once between 1 January and 31 December 2025. These *ad hoc* reviewers do a significant amount of work for the journal, and we appreciate you all. What I find most inspiring about these *ad hoc* reviewers is that they include former editorial board members (whom we call “retired” but they keep working) to early-career researchers. These *ad hoc* reviewers also come from across the globe. These reviewers from across their respective career arcs and geographical locations bring a broad range of perspectives to the reviews, and the journal is better for it. We thank you all!

Hossam Abdelhamed

Ernesto Abel-Santos

Tomaz Accetto

Michael Adams

Adel Talaat

Konstantina Akritidou

Birgit E. Alber

Alessandra M. Albertini

Gladys Alexandre

Pietro Alifano

Lee-Ann H. Allen

Thorsten Allers

Samuel Alvarez-Arguedas

Farhan Anwar Khan

Amjad Islam Aqib

Catherine R. Armbruster

Chelsie Elizabeth Armbruster

Sandra K. Armstrong

Michel Arthur

Irina Artsimovitch

Bernard P. Arulanandam

Htin Lin Aung

Naama Aviram

Sheyda Azimi

Paul Babitzke

Taeok Bae

Jinna Bai

Harsh Bais

David P. Ballou

David A. Baltrus

Ehud Banin

Raul G. Barletta

François-Xavier Barre

Thomas Bartlett

Mirko Basen

Anke Becker

Boris R. Belitsky

Kelly S. Bender

Jose A. Bengoechea

Brittany Bennett

Ivan Berg

Thomas G. Bernhardt

Dany Beste

Shantanu Bhatt

Andrew N. Binns

Robert M. Blumenthal

Magdalena Boguta

Jennifer M. Bomberger

Elizabeth Boon

Bradley R. Borlee

Jeffrey L. Bose

Grant R. Bowman

Jeff M. Boyd

Joel Bozue

Marc Bramkamp

Volkmar Braun

Miriam Braunstein

Shaun R. Brinsmade

David W. Britt

Roland Brosch

Eric D. Brown

Sam Brown

Nicole R. Buan

David Burstein

Briana M. Burton

Leah Bushin

Allen R. Buskirk

Mark J. Buttner

Matthew T. Cabeen

Laty Adriella Cahoon

Susana Campoy

Michael G. Caparon

Monica Cartelle Gestal

Clayton C. Caswell

Yunrong Chai

Patricia A. Champion

Josephine R. Chandler

Chung-I Chang

Matthew R. Chapman

Kausik Chattopadhyay

Wagaw Chekole

Joseph Chen

Peter Chien

W. Seth Childers

Rhishita Chourashi

Nicholas P. Cianciotto

Gino Cingolani

Sarah E. Clark

Tom Coenye

Alan J. Collins

Fabian Moritz Commichau

Laurie Comstock

Ciaran Condon

Timothy L. Cover

Lindsey B. Crawford

Cristian Crisan

Mark Cronan

Sean Crosson

Charles J. Czuprynski

Eric Déziel

David Dubnau

Jan-Ulrik Dahl

Kurt Martin Dahlstrom

Antoine Danchin

Ajai A. Dandekar

Charles J. Daniels

Veronique A. Dartois

Andrew J. Darwin

J. Muse Davis

Leslie A Day

Michaël Deghelt

Patrick H. Degnan

Xin Deng

Adam M. Deutschbauer

Neeraj Dhar

Victor J. Dirita

Andreas Diepold

Lars E.P. Dietrich

Joseph P. Dillard

Nicholas A. Dillon

Huangen Ding

Tobias Doerr

William T. Doerrler

Stephen K. Dolan

Huijuan Dong

Tao Dong

Michael S. Donnenberg

Kelly S. Doran

Patricia C. Dos Santos

Simon L. Dove

Andreas Dräger

Kangmin Duan

Anne K. Dunn

Julie C. Dunning Hotopp

Marylise Duperthuy

Dipak Dutta

Jonathan Dworkin

Michelle Dziejman

Andrew Edwards

Sabine Ehrt

Zehava Eichenbaum

Patrick Eichenberger

Marwa A. El-Beltagy

Philip Michael Elks

Marie A. Elliot

Hyungjin Eoh

Marc Erhardt

Jorge C. Escalante-Semerena

Aria Eshraghi

Manuel Espinosa-Urgel

Robert Fagan

Melinda J. Faulkner

Heather Feaga

James Gregory Ferry

Paul D. Fey

Sergio Raposo Filipe

Steven E. Finkel

Francisco J. Florencio

Gilberto E. Flores

Josué Flores-Kim

Kevin Forsberg

Timothy J. Foster

Casey C. Fowler

Elizabeth M. Fozo

Matthew S. Francis

Dara W. Frank

Gad Frankel

Scott G. Franzblau

Nancy E. Freitag

Shinsuke Fujiwara

Friedrich Götz

Jean-Marc GHIGO

Devarshi Gajjar

Anne Galinier

Jose Luis Garcia

Michael J. Gebhardt

Otto Geiger

Abhrajyoti Ghosh

Karine A. Gibbs

Rosario Gil

Nicole M. Gilbert

Michael S. Gilmore

Zemer Gitai

Erin Gloag

Mark Gomelsky

Eunhye Goo

Andrew L. Goodman

Marcin Grabowicz

Joerg Graf

Jeffrey A. Gralnick

Christophe Grangeasse

E. Peter Greenberg

Steven T. Gregory

Julia Grimwade

Alan D. Grossman

Anne Grove

Alexandra Gruss

Kirsten Raquel Guckes

Eyal Gur

James Gurney

Ted Hackstadt

Adeline Hajjar

Brian K. Hammer

Lynn E. Hancock

Jo Handelsman

Cara H. Haney

Janelle M. Hare

Joe Harrison

Rasika M. Harshey

Jörg S. Hartig

Graham F. Hatfull

Asma Hatoum-Aslan

Vasili Hauryliuk

Robert P. Hausinger

Christine Heilmann

David R. Hendrixson

Tina M. Henkin

Christophe Herman

Eduardo Pedro Hernandez Ortega

David M. Hershey

Anat A. Herskovits

Penelope Higgs

Hidetoshi Higuchi

N. Luisa Hiller

Rebecca Hinrichs

Deborah Hinton

Brian T. Ho

Anders Hofer

Dawn E. Holmes

Michio Homma

David C. Hooper

Alexander Horswill

Damon Huber

Elton Paul Hudson

Kelly Hughes

Ryan C. Hunter

Ahmed Hussein

Tuanh N. Huynh

Kevin Hybiske

Yasushi Imamoto

James Imlay

Thomas R. Ioerger

Masahiro Ito

William R. Jacobs

Babak Javid

Fabrice Jean-Pierre

Paul A. Jensen

Jeremiah Johnson

Dukas Jurenas

Barbara C. Kahl

Justin R. Kaspar

Daniel B. Kearns

Christian Keller

Kristopher J. Kennedy

Linda J. Kenney

Ayesha Khan

Cezar M. Khursigara

Jeffrey D. Klausner

Eric A. Klein

Matthias Koch

Seiji Kojima

Anna Konovalova

Michael Koomey

Günther Koraimann

Sanna Koskiniemi

Jens Kreth

Christopher J. Kristich

Sherry Kuchma

Dhiraj Kumar

Manish Kumar

Brian H. Kvitko

Borden Lacy

Daniel A. Lafontaine

Lefu Lan

Mariana P. Lanfranconi

Matthew B Lawrenz

Beth Lazazzera

Jared Renton Leadbetter

Alysha K. Lee

Chia Y. Lee

Joon-Hee Lee

Christopher Lefèvre

Lars I. Leichert

Cammie Lesser

Celine M Levesque

Chunhao Li

De-Feng Li

Fu-Li Li

Ruichao Li

Yingjie Li

Yuqing Li

Bruno P. Lima

Bingfeng Liu

Zach Lonergan

Ben Long

Tiffany M. Lowe-Power

Carolyn E. Lubner

Joseph Lutkenhaus

Antonio Machado

Cressida Madigan

Shruthi Magesh

Christian Magni

Thien-Fah Mah

Jacob Malone

Shankar Manoharan

Eric Martens

Melissa J. Martin

Ruth C. Massey

Mark J. Mcbride

Kevin S. Mciver

Corinne Mercier

Kevin Militello

Tatiana V. Mishanina

Dominique Missiakas

Tim Miyashiro

Jonathan M. Monk

Rachel Anne Mooney

Yasu S. Morita

Donald A. Morrison

Vladimir L. Motin

Dallas Mould

Dina A. Moustafa

Volker Mueller

Biswarup Mukhopadhyay

Armen Mulkidjanian

Manne Munikumar

Vivek K Mutalik

Christina A. Muzny

Carey Nadell

Satish K. Nair

Shuichi Nakamura

Daisuke Nakane

Beiyan Nan

David Neau

David E. Nelson

Wai-Leung Ng

Dietrich H. Nies

Pablo Ivan Nikel

Aaron Christopher Nolan

Patrice Nordmann

Kouhei Ohnishi

Anil Ojha

Teresa Olczak

Randall J. Olsen

Chee Ooi

Giulia Orazi

Joerg Overhage

Jon E. Paczkowski

Roman Pantucek

Kai Papenfort

Rebecca E. Parales

Tanya Parish

Hyun-Woo Park

Matthew Parsek

Alexander B. Pastora

Tania Paul

Shelley Payne

Melanie M. Pearson

Eveline Peeters

Tong-Tong Pei

José Penades

Karl Perron

Jason M. Peters

Laurent Poirel

Rebecca M. Pollet

Aimee D. Potter

Ute Römling

Paul B. Rainey

Kumaran S. Ramamurthi

Giordano Rampioni

Jolene Ramsey

Matthew Ramsey

David Rasko

Peter Redder

Alejandro Reyes

Anthony R. Richardson

Jeanine Rismondo

Douglas Risser

Rafael Rivilla

Davide Roncarati

Roy Roop II

Silvia Rossbach

Edward G. Ruby

David Z Rudner

Choong-Min Ryu

Ramakrishnan Sitaraman

Álvaro San Millan

Thomas James Santangelo

Yoann G. Santin

Karin Sauer

Gary Sawers

Matthew M. Schaefers

Patrick M. Schlievert

Dirk Schnappinger

Rebecca A. Schomer

Stefan Schulze

Martin Schuster

Cynthia L. Sears

Jessica C. Seeliger

William Self

Peter Setlow

Stephanie Seveau

William M. Shafer

Allyson E. Shea

Scarlet S. Shell

Abhishek Shrivastava

Amit Singh

Varsha Singh

Eric P. Skaar

Jon T. Skare

Mikael Skurnik

Wiep Klaas Smits

Gloria Soberón-Chávez

Holger Sondermann

Joseph A. Sorg

Victor Sourjik

Brady Spencer

Vanessa Sperandio

Jörg Stülke

Apollo Stacy

Nicola R. Stanley-Wall

Ann M. Stevens

George C. Stewart

Ann M Stock

Randy B. Stockbridge

Joshua Strout

Masahiro Suzuki

Rita Tamayo

Minjia Tan

Shumin Tan

Gerald W. Tannock

Herve Tettelin

David G. Thanassi

Martin Thanbichler

Preethi Narayani Thattai Ragunathan

Vinai Chittezham Thomas

Jenny-Lee Thomassin

See-Yeun Ting

Anna D. Tischler

David M. Tobin

Hung Ton-That

Guy Edmund Townsend

Beth Traxler

Anke Treuner-Lange

Paula M. Tribelli

Natalia Tschowri

Boo Shan Tseng

Renée M. Tsolis

Masataka Tsuda

Kürsad Turgay

Gottfried Unden

Raphael Valdivia

Martina Valentini

Miguel A. Valvano

Carin K. Vanderpool

Josh Vermaas

Wim F.J. Vermaas

Patrick Videau

Michelle B. Visser

Perumal Vivekanandan

Lawrence Wackett

Joseph Thomas Wade

Catherine Ann Wakeman

Jennifer N. Walker

Mark J. Walker

Daniel Wall

Danhui Wang

Jue D. Wang

Qiyao Wang

Xiaoxue Wang

Xindan Wang

Matthew Wargo

Nicholas Waterfield

Chris Waters

Mary Weber

E Weber-Ban

David S. Weiss

Cornelia U. Welte

Chris Whitfield

Gregory B. Whitfield

John Whitney

Benjamin E. Wolfe

Matthew C. Wolfgang

Kirsten Wolthers

Daniel J. Wozniak

Ryan Wyllie

Jiawei Xing

Yi Xu

Zhihui Xu

Srujana Samhita S. Yadavalli

Zhaomin Yang

Wearn-Xin Yee

Yasuo Yoshikuni

Ryland F. Young

Jing Yuan

André Zapun

Li Zhang

Jinxin Zhao

Ning-Yi Zhou

Jun Zhu

Igor B. Zhulin

Victor De Lorenzo

Willem M. De Vos

Julia C. Van Kessel

Jan-Peter Van Pijkeren

Bert Van Den Berg

Nicole N. Van Der Wel

Sven Vanteeffelen

Chenggang Wu

